# Confounding Factors Affecting the Emotional Intelligence Amongst Jordanian Nursing and Midwifery Undergraduate Students During the COVID-19 Pandemic's Outbreak: A Cross-Sectional Study Using USMEQ-i

**DOI:** 10.3389/fpsyg.2021.770261

**Published:** 2021-10-29

**Authors:** Rafi Alnjadat, Ahmad Al-Rawashdeh

**Affiliations:** ^1^Department of Applied Sciences, Irbid University College, Al-Balqa Applied University, Al-Salt, Jordan; ^2^Nursing Department, Princess Aisha Bint Al-Hussein College for Nursing and Health Sciences Al-Hussein Bin Talal University, Ma'an, Jordan

**Keywords:** emotional intelligence, Jordanian nursing students, COVID-19 outbreak, COVID-19 lockdown, USMEQ-i

## Abstract

**Objective:** This aim of this study was to determine which variables from the demographic data most affect the EI regarding the COVID-19 outbreak and the lockdown amongst the nursing and midwifery students in Jordan.

**Background:** Emotional intelligence (EI) is the ability to recognize, express, comprehend, motivate, influence and regulate emotions proposed the first EI model, which includes three constructs: emotion assessment and expression, emotion consumption and emotion control. During the COVID-19 outbreak and lockdown, face-to-face study methods have been replaced by online teaching, which has caused many psychological effects.

**Method:** A cross-sectional approach was used to measure EI for nursing and midwifery students. The tool was completed online by nursing and midwifery students using Google Forms. All of the findings were received online and then analyzed accordingly. In this study, USMEQ-i was used to gather data from the participants.

**Results:** The general EI score for the student respondents falls into the average score (*M* = 39.6). Regarding the difference between males and females, the results showed no significant difference. Moreover, the general linear regressions analysis of independent variables on EI score showed four significant factors. Nursing students who study in Years 1 and 4 had significantly higher EI scores than those in Years 2 and 3. EI ability decreased when a student's economic status changed from luxurious to middle income. Moreover, an increase in age significantly decreased the value of EI. This study indicates that all nursing and midwifery students who enrolled in general nursing program tended to have higher EI scores than others (*p* = 0.006).

**Conclusion:** Year of study, age, average lifestyle and enrollment in a nursing program were found to be the most significant factors associated with EI amongst Jordanian nursing and midwifery students. This issue needs to be researched further, such that appropriate steps can be taken to address it.

## Introduction

Emotional intelligence (EI) is the ability to recognize, express, comprehend, motivate, influence and regulate emotions (Mayer and Salovey, 1997; Goleman, 1998a,b; Mayer, 2005). Salovey and Mayer (1990) proposed the first EI model, which includes three constructs: emotion assessment and expression, emotion consumption and emotion control. They described EI as “a type of social intelligence that entails the ability to track one's own and others' feelings and emotions, to distinguish between them and to use this knowledge to direct one's thought and behaviour” (Salovey and Mayer, 1990). Therefore, a person with a high EI can communicate more effectively, reduce anxiety and stress, settle disputes, strengthen relationships, sympathize with others, and overcome life's obstacles. EI has an impact on people's lives because it has an impact on human behavior and relationships. More emotionally intelligent students can better control their emotions and empathize with others around them. This can help them enhance their self-motivation and communication skills, both of which are necessary for pupils to become more confident learners. Students who lack emotional intelligence, on the other hand, may get disengaged from school, which will have an adverse effect on their academic achievement (Kant, 2019; Sánchez-Álvarez et al., 2020).

The illness caused by the COVID-19 coronavirus has had a worldwide effect never seen before. Many studies highlighted the detrimental psychological impact of COVID-19 on society which includes people's severe suffering and cognitive dissonance (Saladino et al., 2020; Testoni et al., 2021). The most susceptible members of the community, such as teenagers and students, may suffer mental health repercussions as a result of the confinement used to contain the epidemic (Liébana-Presa et al., 2020).

EI has two primary constructs based on different models, namely, personal and social competency. These constructs are related to the modern results described by Arifin et al. (2012) and Gupta and Bajaj (2018). The capability to fully understand one's own and others' internal states, desires, resources and intuitions, as well as their consequences, is referred to as social competence (Yusoff et al., 2010; Arifin et al., 2012). Personal competence is described as the ability to maintain self-control, over reactive emotions and impulsive thoughts, as well as the ability to foster and direct emotional impulses toward desired outcomes, and the ability to align and collaborate with others in a community or organization to accomplish mutual goals (Yusoff et al., 2010; Arifin et al., 2012).

Regarding social behavior, EI has a positive relationship with communication skills (Austin et al., 2007), with empathy (Austin et al., 2005) and with group emotional competence (Amundson, 2005). EI also has a positive relationship with coping ability (Humphreys et al., 2005). McCallin and Bamford (2007) found that team members need EI to work effectively with their work partners, and low EI leads to poor teamwork, high anxiety level and poor job satisfaction. Conversely, Oginska-Bulik (2005) found a negative relationship exists between perceived stress in work settings and EI and that depression has a negative relationship with EI.

The present study aims to determine which variables from the demographic data most affect the EI regarding the COVID-19 outbreak and the lockdown amongst the nursing and midwifery students in Jordan. In this current study, the EI covers seven domains (Emotional Control, Emotional awareness, Emotional Conscientiousness, Emotional Commitment, Emotional Fortitude, Emotional maturity, and Emotional Expression), (Yusoff et al., 2010). Face-to-face study methods have been replaced by online teaching (Khraisat et al., 2020), which has caused many psychological effects (Nishimura et al., 2021).

### Confounding Factors Affecting EI

#### Personal Factors

Many psychologists have realized the importance of genetics and the environment in assessing intelligence (Brackett et al., 2011). Personal factors, such as gender, ethnicity and hometown location may affect the EI level of individuals (Khraisat et al., 2015). The relationship between the variables of EI is crucial in understanding EI and the factors affecting it. Most studies have considered age and gender as the most influencers of EI (Kuss and Griffiths, 2017; Lewis et al., 2017; Wilson, 2020).

#### Family Factors

Family influences are one of the most critical aspects of demographical contexts that influence EI. Parents have the most direct influence on their children's EI. Kaur and Jaswal (2005) proposed that a significant relationship exists between family and EI. The degree of EI is often influenced by the family's economic situation (Wahyudi, 2018). According to some reports, having a higher economic status correlates with having a higher EI score (Harrod and Scheer, 2005).

#### Academic Factors

Previous research on the connection between EI and academic success has shown conflicting findings (Thomas and Allen, 2021). Years of education, bachelor's or diploma, varieties of curriculums used and new instructional methodologies used within the university or school are all academic factors (Tapia et al., 2006). Many studies have found that EI has a positive effect on academic achievement (Aithal et al., 2016; AkbariLakeh et al., 2018; Astatke, 2018; Jan and Anwar, 2019; Johar et al., 2019; Rabha and Saikia, 2019; Li, 2020).

#### General Effects of EI

On the basis of a large body of research, EI is a determinant of performance in many organizational environments (Habibah et al., 2007; Alam and Ahmad, 2018). Emotionally intelligent doctors can sustain a positive patient–doctor relationship, show more understanding, communicate effectively and collaborate as team members (Khraisat et al., 2015; Ravikumar et al., 2017). There has been considerable research used on topics of workplace performance and social relations. EI has been found to have a positive relationship with the scale and content of social networks (Tang et al., 2020); strong partnerships, visible family care and fewer crises with close friends (Stein, 2017); and greater optimism, stronger appreciation of others' emotions and positions in social contexts, improved public speaking skills and greater marital fulfilment and performance (Singh and Gosain, 2021). EI has also been negatively connected to opioid and alcohol abuse, deflector activity and a strained relationship with groups (González Yubero et al., 2019); and absenteeism with no justification, insularity from learning regions and desolation (Dobrushina et al., 2020).

A growing body of evidence suggests that EI forecasts significant results in various fields (Mayer et al., 2008). In terms of improved social relationships for children, children's EI reliably forecasts promising social and academic results (MacCann et al., 2020), and the ability of children to regulate their emotions tends to affect their social well-being (MacCann et al., 2020). In terms of better social relationships for adults, Obeid et al. (2021) found that adults with higher EI have higher self-perceived competence in responding to their mates' life activities and have a higher self-perception of social ability and useless destructive interpersonal tactics. Individuals with high EI are perceived more positively by others; people view people with high EI as more fun to be around, empathic and compassionate, and more socially adept than those low in EI (Gupta and Bajaj, 2018). In terms of better family and intimate relationships, EI is linked to certain elements of family and personal relationships (Bucich and MacCann, 2019). Regarding better academic achievement, Karthigeyan and Nirmala (2012) stated that EI is associated with higher academic achievement as recorded by teachers. EI also aids academic achievement by increasing the desire to make the most of one's analytical abilities (Sadipour et al., 2017). “The degree to which men use their skills differs rather than the overall total of their abilities” (Hargittai et al., 2019). In terms of better social relationships during work, EI is linked to better relationship results and negotiation outcomes and greater workplace success (Allen et al., 2020). Other findings by Mayer et al. (2008) in relation to high EI are better psychological well-being, better performance in the medical context, better doctor–patient relationship, increased empathy, increased teamwork and communication skills, and increased stress management and organizational commitment abilities.

## Methods

The primary purpose of this study was to determine the EI level for the nursing and midwifery students in Jordan and identify confounding factors from the demographic data most affect the EI during the COVID-19 outbreak and the lockdown.

### Study Design and Participants

This study used a cross-sectional approach to measure EI amongst Jordanian nursing and midwifery students during the COVID-19 lockdown. The reference population in this current study was all the nursing and midwifery students regardless year of study, or the university. In this current study, the student was defined as any individual above 18 years who is enrolled in a recognized nursing or midwifery program in Jordan by ministry of higher education. The tool was completed online by nursing and midwifery students using Google Forms. All of the findings were received online and then analyzed accordingly. The total number of respondents to this current study was 1,100 students.

### Instruments

The questionnaire developed for this study consisted of eight items regarding socio-demographic data of nursing and midwifery students including age, gender, nationality, program name, year of study, CGPA, marital status, and economic status.

In this study, USMEQ-i was used to gather data from the participants. The advantages of this tool are as follows. The questionnaire structure is uniform for all the respondents. The questionnaire is a quick and effective data collection method. The questionnaire is also a valid tool for gathering certain data, as evidenced by data analysis. Collecting data from vast samples and other instruments are complex. USMEQ-i saves time and money. The USMEQ-i used in this study consisted of 13 items related to EI and four items for faking index (Arifin et al., 2012). For all the questions in the inventory, the responses were measured on a Likert scale of 0 to 4 (0 = not like me; 1 = a bit like me; 2 = quite like me; 3 = a lot like me; 4 = totally like me). The USMEQ-i scores were interpreted based on the recommended guideline provided in the USMEQ-i manual (mean domain score: 0–1.20 = low; 1.21–2.80 = average; 2.81–4.00 = high; Yusoff et al., 2010). Consent was considered received from the students by having them fill out the online forms willingly.

#### Validity and Reliability of USMEQ-i

USMEQ-i has been tested and found to have strong construct validity and internal consistency (Yusoff et al., 2010, 2011; Arifin et al., 2012; Yusoff, 2012). Cronbach's alpha values ranged from 0.8 to 0.9 (Yusoff, 2012), indicating high internal consistency.

### Ethical Approval

The studies involving human participants were reviewed and approved the institutional review board at Al-Balqa Applied University which granted the ethical approval for this study (26/3/2/1065).

The participants provided an electronic informed consent, not a written one. The reason for not providing the written informed consent is that the researchers clearly stated in the beginning of the survey that participant's agreement to complete the survey considered as written informed consent and this means that they willingly and freely agree to participate in the study. Survey was created on Google Forms; no physical or face-to-face interviews were done. No legal guardian or next of kin gave an informed consent because age for inclusion in the study was 18 years and above.

### Data Analysis

SPSS data sheet was used to enter the completed forms. The data were double-checked for completeness and cleaned for missing information. SPSS version 27 was used to analyse the data. For categorical variables, demographic characteristics of respondents were defined in terms of frequencies and percentages. General linear model (GLM) was used to check which demographic variables have the greatest influence on EI. The GLM was used to describe the relationship of several independent variables to USMEQ-I score. Moreover, it allows the researchers to identify the predictors factors of a continuous dependent variables by controlling the confounders. It also determines the relationship strength of each independent variable. A *p*-value ≤ 0.05 was considered as the significance level. Prior to the study, the assumptions of each mathematical evaluation were double-checked.

## Results

This study aimed to determine the factors affecting the emotional intelligence amongst Jordanian nursing and midwifery undergraduate students during the COVID-19 pandemic's outbreak. The researchers used an online questionnaire using the USMEQ-i tool. Year of study, age, average lifestyle and enrollment in a nursing program were found to be the most significant factors affecting the EI score.

A total of 1,100 subjects met the inclusion criteria and were identified as respondents for this study. The data below describes their socio-demographic profile.

The demographic data were analyzed using descriptive analysis, and the results are shown in Table 1. The majority of respondents were female (*N* = 870; 79.1%) while the age group was mainly 21 years or more (*N* = 616; 56.0%). The majority of surveyed students were single (*N* = 901; 81.9%). More than two-thirds of the surveyed students had excellent or very good CGPA (*N* = 797). It was observed that the majority of the students were living a middle lifestyle. Regarding the year of study, it was found that all the students were almost equally distributed between year 2 and year 4. However, number of students from year 1 was the highest. A limited number of students was non-Jordanians (*N* = 39; 3.5%).

**Table 1:** Demographic data analysis.

**Variable**	**Frequency (%)**	**Variable**	**Frequency (%)**
**Gender**		**Age**	
Male	230 (20.9)	21 or more	616 (56.0)
Female	870 (79.1)	20 or less	484 (44.0)
**Program**		**Economic status**	
General nursing	653 (59.4)	Luxury life	58 (5.3)
Midwifery	184 (16.7)	Middle life	941 (85.5)
Combined (nursing and midwifery)	263 (23.9)	Very poor life	101 (9.2)
**CGPA**		**Marital status**	
Excellent	282 (25.6)	Single	901 (81.9)
Very good	515 (46.8)	Married	182 (16.5)
Good	273 (24.8)	Divorced	14 (1.3)
Fair	30 (2.7)	Widow	3 (0.3)
**Nationality**		**Academic degree**	
Jordanian	1,061 (96.5)	BSc students	697 (63.4)
International	39 (3.5)	Diploma students	403 (36.6)
**Year of study**			
First year	379 (34.5)		
Second year	228 (20.7)		
Third year	290 (26.4)		
Fourth year	203 (18.5)		

This study showed that the general EI score for the respondents falls into the average score (*M* = 39.6). The general linear regressions analysis of independent variables on EI score showed four significant factors (Table 2). Nursing students who study in Years 1 and 4 had significantly higher EI scores than those in Years 2 and 3. EI ability decreased when a student's economic status changed from luxurious to middle income. In this current study, economic status was categorized as follows: (low family income of <750 JDs per year, middle family income of 750– <2,900 JDs per year and luxurious family income of 2,900–9,000 JDs). Moreover, an increase in age significantly decreased the value of EI score. This study indicates that all nursing and midwifery students who enrolled in general nursing program tended to have higher EI scores than others, who are enrolled in both the midwifery program and the combined program (Nursing and Midwifery), (*p* = 0.006).

**Table 2:** General linear regressions analysis of independent variables on emotional intelligence.

**Variable**	**Adjusted b (95% CI)**	***t* statistic**	***P*-value**
**Year of study**			
Year four	5.157 (2.702, 7.613)	4.121	<0.001
Year one	2.754 (1.98, 4.409)	3.264	0.001
Program type (nursing)	1.953 (0.559, 3.346)	2.750	0.006
Age	−2.840 (−4.424, −1.256)	−3.518	<0.001
Economic status (middle)	−2.800 (−5.144, −0.456)	−2.344	0.019

*Multiple linear regression: the model reasonably fits well, Model assumptions were met, there were no Interaction and multicollinearity problem*.

## Discussion

This study aims to fill several important gaps in the research on Jordanian nursing and midwifery students. Generally, it seeks to identify the level of EI amongst them and check the association between demographic data and EI. The mean EI score amongst all the participants of this study was 2.33. Based on the primary author of USMEQ-i, this result is considered as average (Yusoff et al., 2010). The study's findings were supported by evidence of EI's beneficial effects on students. Additionally, during the pandemic COVID-19 it has been argued that EI might be utilized as a tool to increase student's happiness, which would lead to better quality of life (Mascia et al., 2020).

On the basis of the general linear regression analysis, Years 1 and 4 have the highest EI score amongst Jordanian nursing and midwifery students whereas students from year 2 and 3 have non-significant EI score. In comparison to these findings, Sharon and Grinberg (2018) found that EI levels increased in Year 2 students. Another study done by Parker et al. (2021) found that EI score increases with age and their study revealed that the EI scores had a reasonably good rank-order stability overtime, and this is consistent with the findings of the current study for year 4 students. Similarly, Chew et al. (2013) discovered that EI is substantially linked with final-year student. In addition to the previously mentioned studies, the findings of this current study are supported by Khraisat et al. (2015), who found that EI level increases over time. The possible explanation for the significant results of EI amongst year 1 students is that the previous studies have been conducted in various contexts, and the present work was conducted during the COVID-19 lockdown, in which the study mode was switched from face-to-face to online learning.

Another aspect that influenced the students' EI levels was their age. The findings indicated that students aged 21 or more have a higher EI level than others. The findings of this study support those of Chen et al. (2016) as well as Gardner and Lambert (2019), who found an important positive association between EI and age. The authors were unable to find numerous studies that contradict this result except for one study done by Rappold (2017), which revealed no statistical significance of EI scores amongst different level of students.

The results showed that nursing students have higher EI levels than students from midwifery and other specialties, which is consistent with the study of Sharon and Grinberg (2018), who indicated that EI level has a positive relationship with nursing students' progress in their studies. Many possible causes of this variance can be said to have this effect. One cause is that the nursing curriculum encompasses courses from many different sciences, including biology, chemistry, social sciences and medicine; this supports the conclusion of a study that was done by Sánchez-Ruiz et al. (2010) who found that the curriculum has a significant effect on the sub-dimensions of EI. Other causes include the majority of nursing instructors being females, which can affect nursing students positively in the matter of EI as reported by (Codier et al., 2015; Phipps et al., 2015). In addition, the mode of study in nursing is mixed, that is, male and female students study together in the field of nursing, whereas midwifery students are all females; the presence of students of other sex can positively affect the levels of EI. This is congruent with the findings of Meshkat and Nejati (2017) who reported that females scored higher EI than males; this has a positive effect on males in that they will try to appear more emotionally intelligent in front of their female colleagues. Moreover, nursing is a 4-year program, whereas midwifery is a 2-year program. Lastly, the number of respondents from nursing students was 653, whereas midwifery respondents were only one-third of this number. This factor could be another explanation of the higher EI amongst nursing students compared with midwifery students. Back to the effect of nursing curriculum on EI, the findings of the current study are incongruent with the results of Rappold (2017), who found that nursing curriculum does not improve EI.

Furthermore, the results showed that the average economic status of the students affects the EI level, in terms of luxurious or poor lifestyle. The results are consistent with a number of studies which found that EI is strongly associated with socioeconomic status (Takeuchi et al., 2019; Rajesh et al., 2021; Schmalor and Heine, 2021). The stability of students' economic status influences EI positively. However, students with low socioeconomic status had higher levels of emotional intelligence than those with higher socioeconomic status, according to the findings of (Boakye, 2017).

## Strengths and Limitations of The Study

This current study is not without limitations. Firstly, it was conducted only amongst Jordanian students. Secondly, a cross-sectional design was used instead of the cohort to measure the EI score during the times of the pandemic of COVID-19 during which lockdown was imposed by the higher authorities worldwide and Jordan is not an exception. Therefore, clinical practicum of students was negatively affected as the students were unable to attend clinical settings. However, the researchers managed to get a large sample size, which has a positive impact on the generalisability of the study findings.

## Conclusion

In conclusion, years of study, age, average lifestyle and enrolment in a nursing program were found to be the most significant factors associated with EI amongst Jordanian nursing and midwifery students. This issue needs to be researched further, such that appropriate steps can be taken to address it.

## Data Availability Statement

The raw data supporting the conclusions of this article will be made available by the authors, without undue reservation.

## Ethics Statement

The institutional review board at Al-Balqa Applied University granted Ethical approval for this report, which was given the code 26/3/2/1065. The participants provided an electronic informed consent.

## Author Contributions

RA and AA-R contributed to conception and design of the study, and wrote sections of the manuscript. RA organized the database and performed the statistical analysis. AA-R wrote the first draft of the manuscript. Both authors contributed to manuscript revision, read, and approved the submitted version.

## Conflict of Interest

The authors declare that the research was conducted in the absence of any commercial or financial relationships that could be construed as a potential conflict of interest.

## Publisher's Note

All claims expressed in this article are solely those of the authors and do not necessarily represent those of their affiliated organizations, or those of the publisher, the editors and the reviewers. Any product that may be evaluated in this article, or claim that may be made by its manufacturer, is not guaranteed or endorsed by the publisher.
